# Promoting Motor Variability During Robotic Assistance Enhances Motor Learning of Dynamic Tasks

**DOI:** 10.3389/fnins.2020.600059

**Published:** 2021-02-02

**Authors:** Özhan Özen, Karin A. Buetler, Laura Marchal-Crespo

**Affiliations:** ^1^Motor Learning and Neurorehabilitation Laboratory, ARTORG Center for Biomedical Engineering Research, University of Bern, Bern, Switzerland; ^2^Department of Cognitive Robotics, Delft University of Technology, Delft, Netherlands

**Keywords:** motor learning, neurorehabilitation, robotic assistance, variability, effort, haptic rendering, model predictive controllers

## Abstract

Despite recent advances in robot-assisted training, the benefits of haptic guidance on motor (re)learning are still limited. While haptic guidance may increase task performance during training, it may also decrease participants' effort and interfere with the perception of the environment dynamics, hindering somatosensory information crucial for motor learning. Importantly, haptic guidance limits motor variability, a factor considered essential for learning. We propose that Model Predictive Controllers (MPC) might be good alternatives to haptic guidance since they minimize the assisting forces and promote motor variability during training. We conducted a study with 40 healthy participants to investigate the effectiveness of MPCs on learning a dynamic task. The task consisted of swinging a virtual pendulum to hit incoming targets with the pendulum ball. The environment was haptically rendered using a Delta robot. We designed two MPCs: the first MPC—end-effector MPC—applied the optimal assisting forces on the end-effector. A second MPC—ball MPC—applied its forces on the virtual pendulum ball to further reduce the assisting forces. The participants' performance during training and learning at short- and long-term retention tests were compared to a control group who trained without assistance, and a group that trained with conventional haptic guidance. We hypothesized that the end-effector MPC would promote motor variability and minimize the assisting forces during training, and thus, promote learning. Moreover, we hypothesized that the ball MPC would enhance the performance and motivation during training but limit the motor variability and sense of agency (i.e., the feeling of having control over their movements), and therefore, limit learning. We found that the MPCs reduce the assisting forces compared to haptic guidance. Training with the end-effector MPC increases the movement variability and does not hinder the pendulum swing variability during training, ultimately enhancing the learning of the task dynamics compared to the other groups. Finally, we observed that increases in the sense of agency seemed to be associated with learning when training with the end-effector MPC. In conclusion, training with MPCs enhances motor learning of tasks with complex dynamics and are promising strategies to improve robotic training outcomes in neurological patients.

## 1. Introduction

Robotic devices provide new possibilities for understanding and accelerating motor (re)learning (Lum et al., 2012; Williams and Carnahan, 2014). One of the most common robotic assistance methods employed in motor (re)learning studies is haptic guidance—i.e., physically guide the participants' limbs, e.g., with PD controllers, through a pre-calculated “ideal” movement trajectory (Marchal-Crespo and Reinkensmeyer, 2009). Although haptic guidance was found to enhance learning in low-skilled participants and in rather simple and artificial tasks that do not incorporate task dynamics (Marchal-Crespo et al., 2010, 2013), in more skilled participants and in tasks that require learning the tasks' dynamics, haptic guidance was found to hamper learning (Powell and O'Malley, 2012; Marchal-Crespo et al., 2015). However, an important part of activities of daily living consists of manipulating objects with complex (non-linear and under-actuated) dynamics, for example, carrying a cup of coffee or watering plants (Mayer and Krechetnikov, 2012). We hypothesize that the problem associated with haptic guidance might be twofold: first, participants might rely on the robotic assistance, which in turn limits the perception of the dynamics of the training environment (Powell and O'Malley, 2012; Pezent et al., 2019), and secondly, by enforcing a predefined trajectory, haptic guidance limits motor variability, crucial in motor learning (Wu et al., 2014).

Interacting with complex dynamical systems relies on somatosensory (i.e., proprioceptive and tactile) information (Milner et al., 2007) to convey essential information for fine motor control, such as carrying a virtual cup of coffee (Hasson et al., 2012), tasks that require identifying the natural oscillation frequency of an object (Huang et al., 2007), or learning to manipulate non-rigid objects (Danion et al., 2012). Importantly, manipulating objects with complex dynamics, compared to simple dynamics, revealed stronger activation in brain areas associated with the processing of somatosensory information and the formation of internal models—namely the cerebellum (Milner et al., 2007). Therefore, enhancement of somatosensory information through haptic rendering of virtual environments during robotic training might elicit better motor (re)learning of complex dynamic tasks (Gassert and Dietz, 2018). However, applying assisting forces through haptic guidance in dynamic-dependent tasks might deteriorate the received somatosensory information, and therefore, hamper learning (Powell and O'Malley, 2012). Furthermore, training with haptic guidance was shown to decrease participants' physical effort (Marchal-Crespo and Reinkensmeyer, 2008; Reinkensmeyer et al., 2009), crucial to promote neuroplasticity (Cramer et al., 2011). The assisting forces may even be perceived as disturbance by skilled participants (Marchal-Crespo et al., 2015), hindering the participants' sense of agency—i.e., the feeling of having control over their own movements (Endo et al., 2020). Therefore, it would be beneficial to develop new robotic strategies that minimize the assisting forces to prevent slacking and reduce the interference with the haptic rendering, while still allowing participants to achieve high task performance to promote motivation (Saemi et al., 2012; Widmer et al., 2016).

The second limitation associated with haptic guidance is that by physically constraining the movement to an ideal, yet fixed, trajectory reduces motor variability—i.e., the trial-to-trial variability in muscle activation while performing a motor task (Duarte and Reinkensmeyer, 2015; Ivanova et al., 2020). While motor variability was initially thought to be an undesired product of neuromotor noise, indicating low expertise in the motor task (Harris and Wolpert, 1998), recent literature suggests that it is a desired feature on which the sensorimotor system relies to operate and learn (Wu et al., 2014; Dhawale et al., 2017). While it may be straightforward and intuitive to quantify the motor variability as the performance variability (e.g., end-point accuracy in reaching tasks), it may be more informative to evaluate the variability of the performed movement—i.e., movement variability—especially in redundant tasks that incorporate multiple movement solutions to achieve the same goal (Singh et al., 2016; Levac et al., 2019). Furthermore, the movement variability with respect to the internal dynamics of under-actuated tasks (e.g., the pendulum swing variability) may play a crucial role in learning complex dynamic tasks (Muller and Sternad, 2004). Therefore, robotic assistance should not only avoid restricting the variability of the performed movement but also the movement variability with respect to the internal dynamics of the task to optimally support the exploration of the task dynamics and promote learning.

We propose that Model Predictive Controllers (MPC) might be a good alternative to haptic guidance in order to minimize the assisting forces and allow (and even promote) motor variability during training. An MPC is an optimal control method that employs the dynamical model of the environment to predict the future states of the system and to minimize the assisting forces (Morari and Lee, 1999). The optimization of MPC is redone at each time point to increase the controller performance under unpredictable disturbances, such as the interaction with humans. The online optimal nature of MPCs results in flexible movement trajectories that may promote task exploration while still providing sufficient assistance to perform the task.

**Figure 1:**
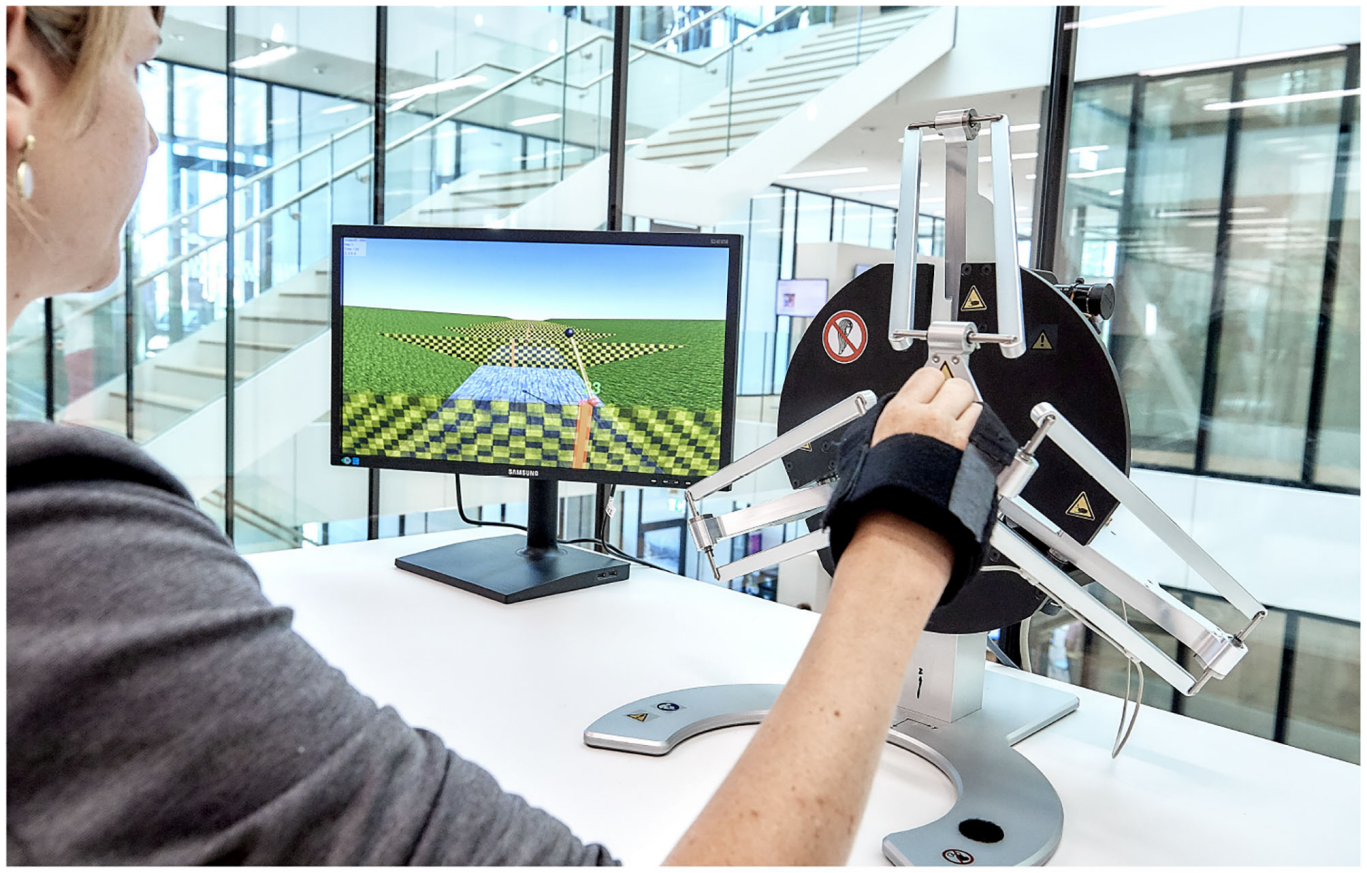
Experimental setup with Delta.3 robot (Force Dimension, Switzerland). The photograph was taken by Adrian Moser for ARTORG Center.

We conducted a between-subject study with 40 healthy young participants to investigate the effectiveness of using MPCs as assistance strategies during learning of a complex dynamic task. The task consisted of swinging a virtual pendulum to hit incoming targets with the pendulum ball, which was haptically rendered on a Delta robot (Force Dimension, Switzerland, Figure 1). The participants could move the pendulum pivoting point by moving the robot end-effector (EE) in a vertical plane to indirectly control the pendulum ball through a rigid rod. Mastering the pendulum task requires the precise manipulation of its states, which have chaotic behavior, and familiarization with its under-actuated non-linear dynamics, similar to holding and moving a cup of coffee without spilling it (Mayer and Krechetnikov, 2012). The challenge was further increased by selecting the positions of the incoming targets to promote movements with swinging frequencies away from the natural oscillation frequency of the pendulum.

We designed two MPCs that differ in terms of the application point of the assisting forces. A first MPC—end-effector MPC—applied the optimal assisting forces directly on the robot end-effector (i.e., the pendulum pivoting point). We developed a second MPC—ball MPC—that applied its forces directly on the virtual pendulum ball. Applying the assistance on the pendulum ball has a direct impact on the pendulum dynamics and is less prone to be affected by the human-robot interaction forces, and thus, only small assisting forces are needed to reach good task performance (Özen et al., 2019). The participants still felt the projected forces from the pendulum ball in the robot end-effector. However, the indirect application of the assisting forces might degrade the perception of the pendulum dynamics and reduce the participants' sense of agency (Özen et al., 2019), which, in turn, might hamper motor learning.

The participants' performance during training and learning at immediate and delayed retention tests were evaluated and compared to a control group that trained without assistance and a group that trained with a conventional haptic guidance controller (PD controller) with fixed trajectories. The participants' task performance was evaluated using measurements of target hitting success, pendulum swing frequency, and three different measures of motor variability: (i) performance variability, i.e., variability in the task score, normally associated to low expertise in the motor task (Harris and Wolpert, 1998), (ii) movement variability, i.e., the variability of the participant's movement, a desired feature that might promote motor learning (Dhawale et al., 2017), and (iii) pendulum swing variability, i.e., the variability of the pendulum angle (internal degree-of-freedom), which might be especially important to promote the exploration of the task dynamics (Muller and Sternad, 2004). We also evaluated the effect of the different controllers on subjective measures of motivation and sense of agency. Transfer of learning was evaluated in a transfer task (inverting the pendulum) with the same pendulum dynamics.

We hypothesized that participants in the end-effector MPC group would reach comparable hitting performance to the haptic guidance group during training and would enhance the movement variability and not hamper the pendulum swing variability compared to the other assisting training strategies. We further hypothesized that training with the end-effector MPC would enhance motor learning compared to the other training strategies. We expected that the superior learning associated with the end-effector MPC would be more evident in the performance metrics reflecting learning of the task dynamics (e.g., higher deviation from the pendulum natural frequency). Moreover, we expected that applying the assisting forces on the pendulum ball would enhance participants' performance and motivation during training, but limit their movement variability and sense of agency, and ultimately, hamper motor learning. Finally, we expected a positive association between the sense of agency and learning.

## 2. Materials and Methods

### 2.1. Experimental Setup

A Delta.3 robot (Force Dimension, Switzerland) was used as a haptic interface in the study (Figure 1). The robot was positioned on a table next to an LCD screen where the visual elements of the game were presented. The robot motion control was implemented in C++ and run at 4 kHz. The game visuals were implemented using Unity (Unity Technologies, US).

**Figure 2:**
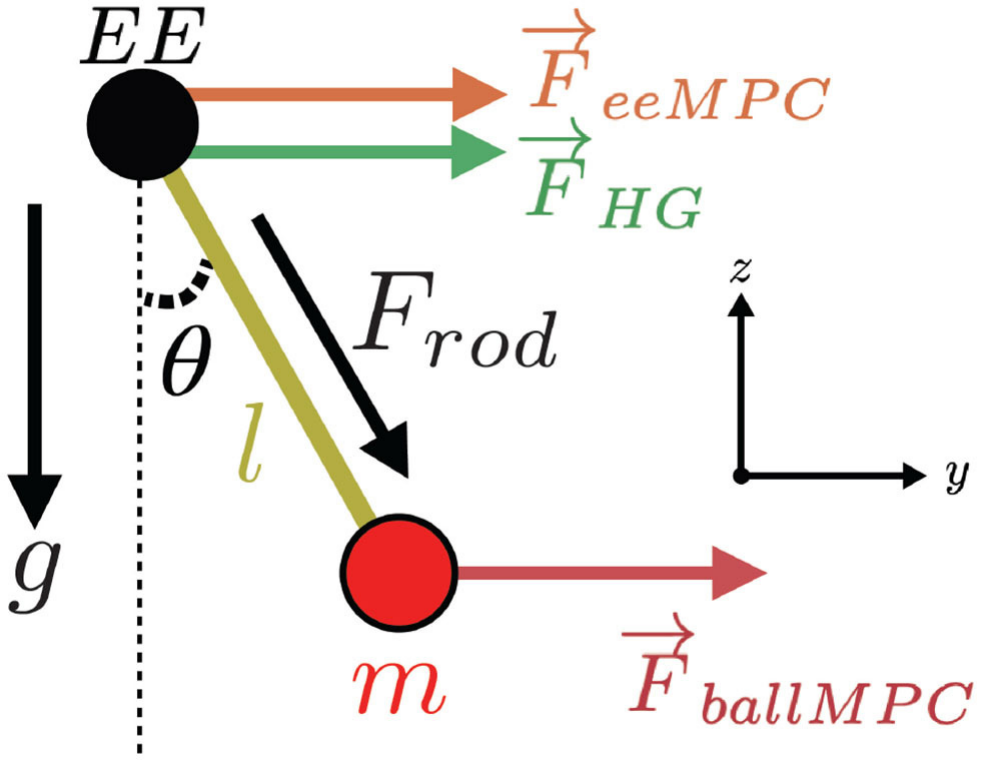
Pendulum dynamics and assisting forces from the haptic guidance controller (*F*_*HG*_), end effector MPC (*F*_*eeMPC*_), and ball MPC (*F*_*ballMPC*_).

The experiment required controlling the motion of a pendulum in the virtual environment. By moving the robot end-effector in the vertical plane, participants controlled the motion of the pendulum pivoting point (a black ball, Figure 2) and indirectly the pendulum mass (red ball). Participants could feel the forces from the pendulum through the robot end-effector (haptic rendering).

### 2.2. Pendulum Dynamics

The implementation of the pendulum dynamics was described in detail in Özen et al. (2019). Here, only a brief summary is given for completeness.

The pendulum was constrained to a vertical plane with a stiff PD controller, allowing for two degrees-of-freedom (DoF) movements in *y* (horizontal axis) and *z* (vertical axis, Figure 2). The pendulum internal DoF (θ) is defined with respect to its pivoting point. The pendulum mass *m* and rod length *l* were set to 0.6 kg and 0.25 m, respectively. The gravity coefficient *g* was set to 1/3 of the real earth gravity and the damping coefficient *c* was set very low (3*e*^−6^ N.s/rad) to prevent the pendulum to stabilize itself and to keep the task challenging. These values result in a pendulum natural frequency (i.e., how the pendulum angle, θ, oscillates when uninterrupted) of 0.57 Hz.

The equation of motion that rules the swing of the pendulum ball (θ) based on the robot end-effector movement (*y*, *z*) is of the form:

(1) $$\ddot{\theta }=-\frac{1}{l}\left((\ddot{z}+g)\sin\theta +\ddot{y}\cos\theta \right)-\frac{c}{ml^{2}}\dot{\theta }.$$

The haptic rendering forces transmitted through to the robot EE were calculated with the following equation:

(2) $$F_{rod}=m\left((\ddot{z}+g)\cos\theta -\ddot{y}\sin\theta +{\dot{\theta }}^{2}l\right).$$

### 2.3. The Pendulum Task

**Figure 3:**
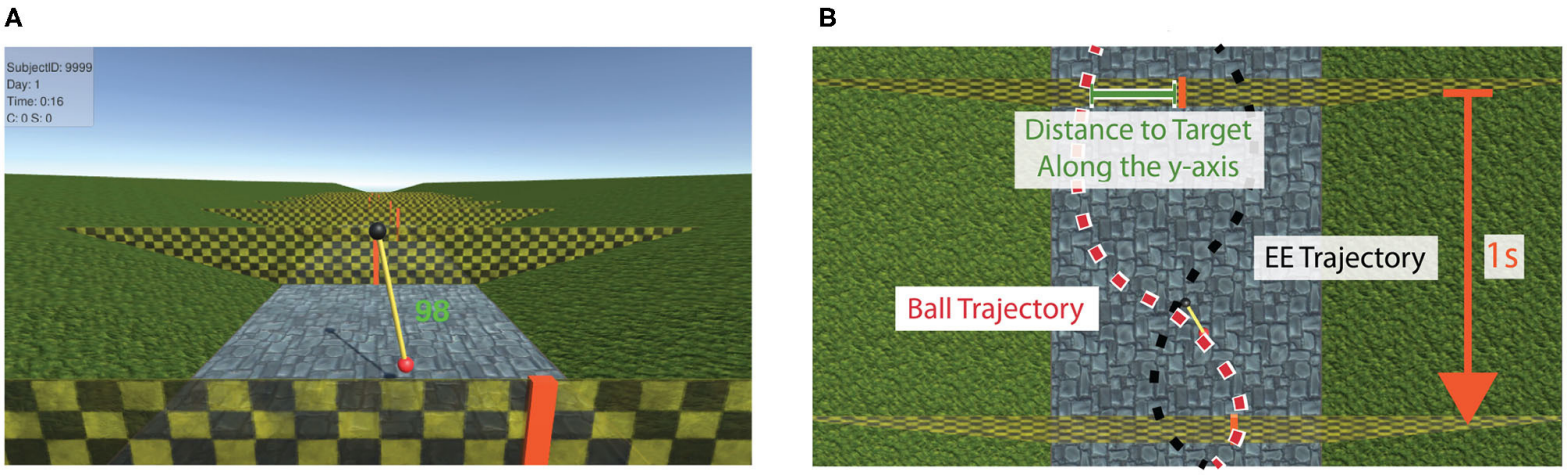
The virtual training environment. **(A)** Participants' point of view. The walls with vertical targets (in red) approach toward the participants with 1 s between walls. The performance score (proportional to the distance to target along the y-axis) is visually presented above the targets for 0.5 s, indicating how close the previous target was hit. **(B)** Top view of two consecutive targets. Example trajectories for the pendulum red ball and the black ball are depicted as red and black dashed lines, respectively.

The task to be learned consisted of moving the pendulum pivoting point (i.e., black ball, Figure 2) in the horizontal and vertical axes to swing the pendulum in a way that the pendulum mass (i.e., red ball, Figure 2) hits vertical targets (i.e., orange vertical lines on walls) moving toward the participants at constant velocity (Figure 3A). There was a 1 s interval between targets. The positions of the targets on the walls were randomly presented either at the center of the screen, 12 cm on the right, or 12 cm on the left. A small amount of random displacement (σ: 1.25 cm) was added to these three mean positions to increase the task difficulty.

Depending on the distance—along the *y*-axis—between the red ball and each target at hitting time (Figure 3B), a score (between 0 and 100) was calculated:

(3) Score=100−Distance(mm)*0.5.

This performance score was visually presented to the participants in green color—or red if score was 0—each time a target was passed, right above the target location (Figure 3A). The score disappeared after 0.5 s.

A second task with the same pendulum dynamics was included to test the transfer of learning. This transfer task consisted of swinging the pendulum from the stable equilibrium point (θ = 0 ° and $\overset{∙}{\theta }=0$ °/s) to invert it and maintain the unstable vertical configuration with the red ball on top (θ = 180 ° and $\overset{∙}{\theta }=0$ °/s). The participants were instructed to keep the pendulum inverted as long as possible. A score—adapted from Smith et al. (2020)—was presented on the screen to provide feedback during the transfer test. The score was reset at the beginning of the test and increased according to the equation:

(4) $$Score_{transfer}=\int_{inv_{begin}}^{inv_{end}}k\;z_{diff}dt,\text{ if }z_{diff}>0.$$

The inversion began (*inv*_*begin*_) each time the height of the pendulum red ball exceeded the height of the pivoting point (*z*_*diff*_ > 0), and ended when it fell below (*inv*_*end*_). The score increased as long as the pendulum was inverted (*z*_*diff*_ > 0), proportional to *z*_*diff*_ and an arbitrary unit-less constant *k*. If the ball fell below the height of the pivoting point (*z*_*diff*_ < 0), the score was retained until a new inversion was achieved.

### 2.4. Training Strategies

Participants trained the pendulum target hitting task with one of four different training strategies: (i) No guidance (Control): only the pendulum dynamics were haptically rendered, (ii) End-effector MPC (eeMPC): the online calculated optimal assisting forces were applied at the end-effector, (iii) Ball MPC (ballMPC): the online calculated optimal assisting forces were applied at the pendulum ball, and (iv) Haptic guidance (HG): fixed-trajectory based assistance was applied at the end-effector. The assisting forces were applied in the *y* axis (Figure 2). We did not provide assistance in the *z* axis as it was sufficient to move the end-effector in the *y* axis to successfully hit the targets. Furthermore, applying assisting forces in the *z* axis would have further masked the perception of the pendulum mass while mostly providing arm weight support. The controllers were implemented using ACADO Toolkit (Houska et al., 2011). For detailed technical information about the controllers, please refer to the Supplementary Material.

The working principle of an MPC is to predict the future states of the system (i.e., the trajectories of the pendulum red ball and end-effector) using a model of the system (i.e., the pendulum's equation of movement), and calculate the control input (i.e., assisting force) according to an optimization problem, where a cost function is designed and minimized. The *Cost* function we designed had the form:

(5) $$\begin{array}{r} \begin{array}{c} Cost=\overset{t+N}{\underset{k=t}{\sum }}h_{k}^{⊤}W_{k}h_{k} \end{array} \end{array}$$

(6) $$\begin{array}{r} h_{k}=\left[\begin{array}{c} \text{Distance to Target Along the }y\text{ Axis}\\ \text{Ball Linear Speed}\\ \text{Assisting Forces} \end{array}\right]\; \end{array}$$

where *t* is the current time, *N* is the predicted horizon length (i.e., how far the future trajectories are considered) and *k* is the time step along the predicted horizon which ranges from *t* to *t* + *N*. The *Cost* function was quadratic in terms of the penalization vector *h*_*k*_, so that it was convex and smooth. Minimizing the *Cost* function corresponded to simultaneously minimizing: the distance to target along the *y* axis—i.e., how far the pendulum ball was to the target in the moment of wall impact—, the speed of the pendulum ball—required to maintain a stable pendulum movement—, and the magnitude of assisting forces. The simultaneous minimization of these three elements was counteractive to each other as the motion of the pendulum could be controlled more accurately with more assisting forces. The optimization trade-off was adjusted dynamically at each time step by the 3*x*3 diagonal weight matrix *W*_*k*_. The weight matrix changed along the predicted horizon in order to keep the assisting forces low while the pendulum plane is far from the walls of the targets, and therefore, increase participants' movement freedom when far from the incoming wall. Specifically, the penalization of the assisting forces was set inversely proportional to the distance from the target plane. Thus, as the targets approached toward the vertical plane of the pendulum, the cost function became more restrictive. The optimization is recalculated at each time step (at 80 Hz), which provides robustness against unpredictable disturbing elements arising from the interaction with the participants.

#### 2.4.1. End-Effector MPC

The assisting forces from the end-effector MPC were applied to the robot end-effector—in the *y* axis (Figure 2). The assisting forces were limited to 8 N. This value was chosen based on previous tests, such that the assisting forces are sufficient to guide a passive participant to accurately hit the targets (Özen et al., 2019).

#### 2.4.2. Ball MPC

The ball MPC was implemented following the same principle as the end-effector MPC, with the difference that the calculated assisting forces were applied at the pendulum ball rather than directly on the robot end-effector. Applying the assisting forces on the pendulum ball had a direct impact on the pendulum swing since participants could only apply forces on the robotic end-effector and could not directly counteract the assisting forces. We set the limit for the controller force to 1 N based on preliminary studies where we observed that this amount was sufficient to achieve high task performance (Özen et al., 2019).

#### 2.4.3. Haptic Guidance

The haptic guidance strategy was implemented as a conventional PD controller (P: 100 N/m.kg, D: 20 N.s/m.kg), which is commonly employed to follow fixed trajectories in robot-assisted therapy (Marchal-Crespo and Reinkensmeyer, 2009). In order to have a fair comparison with the MPC controllers, the desired trajectory was calculated following a similar optimization principle. However, while in eeMPC a new optimization was run at each time step resulting in a new trajectory 80 times per second, the HG trajectory was calculated only once for each target (i.e., right after passing the previous target). A cubic B-spline was fitted to the resulting optimal trajectory and fed in the PD controller. The assisting forces—dependent on the deviation from the fixed trajectory—were applied at the end-effector. To keep the comparison with the end-effector MPC fair, the assisting forces were also limited to a maximum of 8 N.

### 2.5. Study Protocol

The study was approved by the Cantonal Ethics Committee and the Swiss Agency for Therapeutic Products (Swissmedic) and conducted in compliance with the Declaration of Helsinki. Forty-one healthy young participants provided written consent to participate in the study (19 females, age mean: 34, std.: 11). Three participants were left-handed, according to the Waterloo handedness questionnaire (Bryden, 1977). One participant did not finish the study, and therefore, was excluded from data analysis.

**Figure 4:**
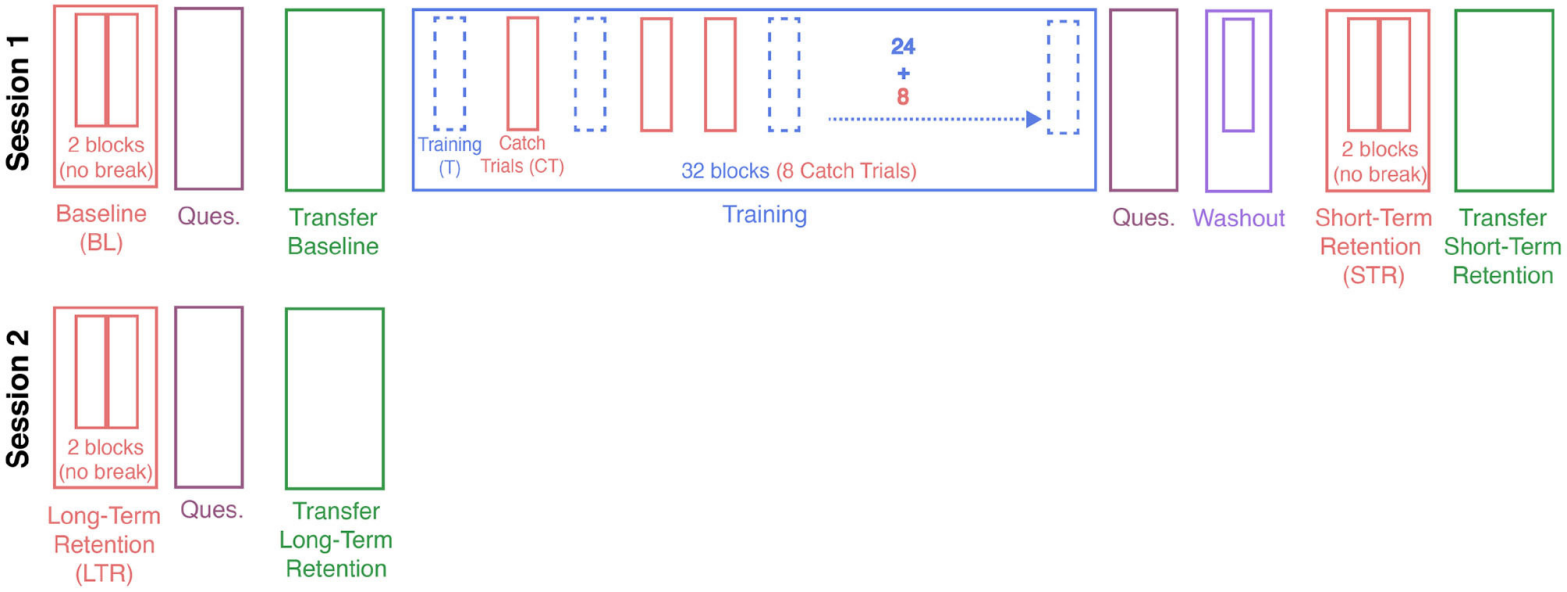
Experimental protocol. Participants were randomly allocated to one of four training groups: Control, End-effector MPC, Ball MPC, and Haptic Guidance. Participants completed two sessions, with 1–3 days between sessions. Training blocks (x32) and catch-trials blocks (x8) were used to analyze the effect of training with different assistance strategies on the metrics considered. Motor learning was evaluated according to changes in the metrics from baseline (BL) to short-term retention (STR) and long-term retention (LTR). Furthermore, transfer learning was evaluated according to changes in the transfer task performance from transfer baseline to transfer short-term retention and long-term retention. After baseline, after training and after long-term retention, participants responded to a selection of statements from the Intrinsic Motivation Inventory (IMI) and agency section of the embodiment questionnaire (Ques.).

Participants were randomly allocated to one of four training groups of ten participants each (between-group design: no guidance [Control], end-effector MPC [eeMPC], ball MPC [ballMPC], and haptic guidance [HG]). Participants were informed about the possibility that the robot could assist them during the task, but did not know in which training group they were assigned. The overview of the study protocol is shown in Figure 4.

The study consisted of two experimental sessions, separated by 1–3 days. At the beginning of the first session, the position and orientation of the robot and the screen were adjusted on the table depending on the handedness of the participant. The dominant hand of the participant was attached to the robot end-effector with Velcro® straps (Figure 1). Participants were instructed to rest the elbow on the table during the whole experiment. The experiment started with verbal/visual instructions about the pendulum and the haptic rendering of its dynamics, followed by a familiarization period (1 min) without assisting forces, when the participants were allowed to move the pendulum to feel its dynamics.

Participants were then instructed about the main task to be learned (i.e., moving the robot end-effector to move and swing the pendulum to hit incoming targets with the pendulum ball). After task instruction, participants performed a baseline test block (with only the haptic rendering of the pendulum dynamics), where they were requested to hit the targets as close as possible. The baseline test included 40 consecutive targets. Participants were then verbally and visually instructed about the transfer task (i.e., inverting the pendulum). The transfer baseline test lasted 1 min.

After a short break, the training blocks for the main task started (with a short reminder about the task goal). There was a total of 32 training blocks, each consisting of 20 targets, with an optional break after the first 16 blocks. The duration of this break was not fixed. During training, the robot assisted participants allocated in the eeMPC, ballMPC, and HG groups while participants in the control group only felt the haptic rendering of the pendulum dynamics. The assistance was unexpectedly removed in eight over the 32 training blocks (catch-trial blocks). The catch-trial blocks acted as a reference (Assistance: OFF) to compare the effects of the assisting forces (Assistance: ON) on the performance metrics. The order of the catch-trial blocks was pseudo-randomized (half before the break) and fixed for all participants.

A non-assisted wash-out block of 20 targets was performed right before the short-term retention test (STR) in order to diminish the potential transient effects of training with the assisting controllers (e.g., “slacking”). The short-term (immediate) retention test followed the same structure as the baseline (i.e., same target location order, no assistance). The first experimental session finished with a transfer test—i.e., participants tried to invert the pendulum for 1 min without assisting forces. The first session of the experiment lasted around 30 min. Participants returned after 1–3 days to perform the long-term (delayed) retention test (LTR) and a second transfer test (with the same structure as on the first session).

The targets' location order was identical for the baseline, short-term, long-term retention test blocks, and catch-trial blocks (baseline, STR and LTR were a repetition of two identical catch-trial blocks). The targets' order in the training blocks was different between blocks, but identical across participants. The average score after each test or training block was visually presented to participants to increase their motivation.

We assessed the participants' subjective motivation and sense of agency after baseline, the last training block, and the long-term retention test (Figure 4). We employed 12 statements (see Supplementary Material) from the well-established Intrinsic Motivation Inventory (IMI, Ryan et al., 1990). We focused on assessing *interest*/*enjoyment*, *perceived* *competence*, *effort*/*importance*, and *pressure*/*tension* (three statements per subscale). Participants ranked their agreement with the statements on a Likert scale between 1 and 7 points; 1 indicated “not at all” and 7 indicated “very true”. The sense of agency was assessed employing three questions from the embodiment questionnaire used in Piryankova et al. (2014). The agency questions were adapted to the pendulum task. A Likert scale between −3 and 3 points was used for ranking; −3 indicated “strongly disagree” and 3 indicated “strongly agree.” The questionnaire was presented in English. Answers from the same questions at different experimental times were always visible, to exclude the effects of the differences in participants' memory skills on the results (Marchal-Crespo et al., 2019). For a complete list of questions, please refer to the Supplementary Material.

### 2.6. Data Processing

Different metrics were selected to evaluate the learning of the task goal (i.e., accuracy in the hitting task), learning to control the pendulum dynamics (i.e., deviation from pendulum natural frequency), performance variability, movement variability and pendulum swing variability.

We selected the *score* (proportional to the deviation between the pendulum ball and the target along the *y* axis at the hitting time, Equation (3), Figure 3) as the main performance metric to evaluate the target **hitting performance** (i.e., accuracy). The metric is similar to the hitting error of Marchal-Crespo et al. (2013), but adapted for our task. The average *score* for each test/block was taken as a data point for the analysis.

In order to evaluate if participants learned how to control the pendulum dynamics, we chose the **deviation from the pendulum natural frequency** as the second performance metric (*PSD % around* ω_*n*_). In order to skillfully hit the targets, participants need to control the motion of the pendulum ball to follow an aperiodic trajectory, since the target locations were randomized and did not follow a periodic order. This required shifting the swing/angle frequency of the pendulum away from its natural frequency—i.e., to compensate the natural periodic swing of the pendulum. In order to quantify this frequency deviation, we first calculated the power spectral density of the pendulum angle data for each test/block. We then calculated how much percentage of the power spectral density was around the pendulum natural frequency (ω_*n*_ = 0.57 Hz), alike in Huang et al. (2007). Since the lowest resolution of our data corresponds to 0.05 *Hz* (the catch-trials were the shortest blocks; 20 s), we selected the bandwidth ω_*n*_±0.05 as the pendulum natural frequency. The percentage value (*PSD % around* ω_*n*_) was calculated for each block for the data analysis.

The way in which the motor variability affects learning was found to be task-specific and dependent on how the variability is quantified (He et al., 2016). One straightforward way to quantify the motor variability is as the **performance variability**, which is expected to reduce with higher expertise (Harris and Wolpert, 1998). Therefore, to evaluate the performance variability (i.e., how variable the *score* was) the standard deviation of the score, *std*(*score*), was calculated for each block. On the other side, the variability of the performed movement was found to drive exploration of task dynamics and therefore motor learning (Wu et al., 2014). To analyze the **movement variability** (i.e., how variable the participants' movements were) the standard deviation of the end-effector horizontal position during each block, *std*(*eePos*), was calculated for each block. However, since the pendulum dynamics are under-actuated, the end-effector movement does not capture the whole aspect of the movement. The variability in the internal movement of a task may be an essential contributor to the exploration of the task dynamics (Muller and Sternad, 2004). The internal movement of our task dynamics is the pendulum swing. Therefore, in order to analyze the **pendulum swing variability**, the standard deviation of the pendulum θ angle, *std*(*Theta*), was calculated for each block.

The absolute **assisting forces** applied by the different controllers at the robot end-effector in the *y* direction—directly on the EE by the eeMPC and HG controllers or projected through the rod from the ballMPC—were compared across training groups. Additionally, in order to evaluate the controllers' behavior during training, the human-robot **interaction forces** between the participants' hands and the robot end-effector were estimated using Reaction Torque Observers (Murakami et al., 1993). Only the interaction forces in the *y* axis were considered since *y* is the axis along which participants move to fulfill the target hitting task.

Finally, we evaluated the **transfer task performance** using the *score*_*transfer*_ variable calculated during the inverted pendulum transfer tests (Equation 4).

### 2.7. Statistical Analysis

In order to evaluate if the random allocation of participants within the four training groups resulted in differences in baseline performance, we compared the baseline data between groups using one-way ANOVA. The association between the hitting task performance (*score*) and the deviation from the pendulum natural frequency (*PSD % around* ω_*n*_) was evaluated with repeated-measures correlation (Bakdash and Marusich, 2017), which accounts for the within-subject dependence of observations. Participants' performance data during baseline, short-/long-term retention, and training blocks were used for this correlation.

We used linear mixed models (LME) to evaluate the effect of the different training strategies (*Group*: Control, eeMPC, ballMPC, and HG), and time on the performance variables. We employed the mean values of each performance variable during each test/block as dependent variables (i.e., each block/test had one data point for each performance metric and participant). We used the *lmerTest* package in *R* (Kuznetsova et al., 2017). The model assumptions (i.e., normality and equal variance) were visually inspected with Q-Q plots and residual vs. fitted value plots for each variable. The *emmeans* package with FDR correction was used for all multiple comparisons (Benjamini and Hochberg, 1995; Benjamini and Yekutieli, 2001). The significance level was set to α = 0.05.

In order to analyze the effect of enabling the robotic assistance on the performance metrics, we analyzed the differences between training blocks with assistance (24 blocks) and blocks where the assistance was unexpectedly removed (8 catch-trial blocks, Figure 4). We note that the control group did not receive assistance during the training blocks. The following LME model was used to analyze the data during training:

(7) $$\begin{array}{r} PM\sim Group*Assistance_{ON/OFF}*Time+\left(1+Time|Subject\right) \end{array}$$

where *PM* represents the analyzed performance metric, *Group* (categorical) corresponds to the different training strategies, and *Assistance*_*ON*/*OFF*_ (categorical) corresponds to the status of the assisting controller (*ON* during training blocks, *OFF* during catch-trials). The *Time* (continuous) corresponds to the order of the block within the Training (Figure 4, from 1 to 32). The *Time* variable was included to take into account the order of the training (T) and catch-trial (CT) blocks since this order was randomized within the Training (but the same for all participants). Finally, a random intercept for the *Subject* and a random slope for the *Time* was included in the model to account for the dependency of the data and account for the variance attributed to the differences in participants' personal abilities. The evaluation of the data was performed as follows:

- In order to assess if the training strategies had an effect on the performance metrics, the effect of *Assistance*_*ON*/*OFF*_ (i.e., robotic assistance *ON* vs. *OFF*) per each training strategy was tested (i.e., comparison of the estimated marginal means of the robot-assisted training blocks and the catch-trial blocks). This resulted in four multiple comparisons (i.e., one per training strategy, Table 1, white rows).
- In order to compare the effects of the different training strategies on the performance metrics, we checked if there was a significant interaction effect between the training group and the controller status (*Group* *x* *Assistance*_*ON*/*OFF*_) with ANOVA tests. *Post-hoc* comparisons were performed if the interaction was significant. This corresponded to six comparisons (Table 1, gray-shaded rows).

**Table 1:** Results from the multiple comparisons tests to evaluate the effects of the training strategies on the performance metrics during training.

**Comparison**	**Interaction forces**	**Assisting forces**	**Hitting performance**	**Deviation from pendulum **ω**_*n*_**	**Performance variability**	**Movement variability**	**Pendulum swing variability**
Control:ON - Control:OFF	**0.004**	1.0	0.106	** <0.001**	0.693	0.953	**<0.001**
eeMPC:ON - eeMPC:OFF	**<0.001**	**<0.001**	**<0.001**	**<0.001**	**<0.001**	**<0.001**	0.115
HG:ON - HG:OFF	**<0.001**	**<0.001**	**<0.001**	**<0.001**	**<0.001**	**<0.001**	**<0.001**
ballMPC:ON - ballMPC:OFF	**<0.001**	0.289	**<0.001**	**<0.001**	**0.001**	0.223	**<0.001**
(Control:ON - Control:OFF) - (eeMPC:ON - eeMPC:OFF)	**<0.001**	**<0.001**	**<0.001**	**<0.001**	**<0.001**	**<0.001**	0.162
(Control:ON - Control:OFF) - (HG:ON - HG:OFF)	**<0.001**	**<0.001**	**<0.001**	**<0.001**	**<0.001**	**<0.001**	**0.002**
(Control:ON - Control:OFF) - (ballMPC:ON - ballMPC:OFF)	**0.002**	0.383	**<0.001**	**<0.001**	**<0.001**	0.419	**0.001**
(eeMPC:ON - eeMPC:OFF) - (HG:ON - HG:OFF)	**<0.001**	**<0.001**	0.095	**0.029**	0.333	0.471	**<0.001**
(eeMPC:ON - eeMPC:OFF) - (ballMPC:ON - ballMPC:OFF)	**<0.001**	**<0.001**	**<0.001**	**<0.001**	**<0.001**	**<0.001**	**<0.001**
(HG:ON - HG:OFF) - (ballMPC:ON - ballMPC:OFF)	**<0.001**	**<0.001**	**<0.001**	**<0.001**	**<0.001**	**<0.001**	0.837

*ON stands for the robot-assisted training blocks (Assistance_ON_), while OFF stands for the catch-trial blocks without assistance (Assistance_OFF_). The white-shaded rows are within-group differences, and gray-shaded areas rows are the interaction contrasts (between-group differences), performed when there was a significant Group x Assistance_ON/OFF_ interaction as a result of ANOVA. Significant p-values are indicated in bold font*.

In order to analyze whether the different training strategies had an effect on short- and long-term learning—for both the main task of hitting targets and the inverted pendulum task (i.e., transfer task)—we employed the following LME model (*Time*: baseline, short-term retention, and long-term retention):

(8) $$\begin{array}{r} PM\sim Group*Time+\left(1|Subject\right). \end{array}$$

The evaluation of the data was done as follows:

- We analyzed whether participants in each training group improved their performance after training (from baseline to short-term retention), and at long term (from baseline to long-term retention), and whether they retained their performance between experimental sessions (from short- to long-term retention). This corresponds to 12 multiple comparisons (three *Time* pairs per training group, Table 2, white rows).
- In order to evaluate whether training with the different training strategies had different effects on the performance changes over time, we checked for interaction effects (*Group* *x* *Time*) with ANOVA tests. *Post-hoc* comparisons were performed if the interaction was significant. This corresponds to 18 pair-wise comparisons (three *Time* pairs and the six *Group* pairs, Table 2, gray-shaded rows).

**Table 2:** Results from the multiple comparisons tests to evaluate the effects of the training strategies on short- and long-term learning.

**Comparison**	**Hitting performance**	**Deviation from pendulum **ω**_*n*_**	**Performance variability**	**Movement variability**	**Pendulum swing variability**	**Transfer task performance**
Control:STR - Control:BL	**<0.001**	0.451	**<0.001**	**<0.001**	0.476	**<0.001**
Control:LTR - Control:BL	**<0.001**	0.848	**<0.001**	**<0.001**	0.844	**<0.001**
Control:LTR - Control:STR	0.823	0.514	0.88	0.461	0.565	0.191
eeMPC:STR - eeMPC:BL	**<0.001**	**<0.001**	**<0.001**	**<0.001**	0.476	**0.036**
eeMPC:LTR - eeMPC:BL	**<0.001**	**<0.001**	**<0.001**	**<0.001**	0.476	**<0.001**
eeMPC:LTR - eeMPC:STR	0.96	0.848	0.88	0.275	0.624	**0.007**
HG:STR - HG:BL	**<0.001**	0.848	**<0.001**	**<0.001**	0.476	**0.006**
HG:LTR - HG:BL	**<0.001**	0.250	**<0.001**	0.275	0.476	**0.013**
HG:LTR - HG:STR	0.96	0.295	0.34	**0.007**	0.844	0.697
ballMPC:STR - ballMPC:BL	**<0.001**	0.848	**<0.001**	0.082	0.476	0.078
ballMPC:LTR - ballMPC:BL	**<0.001**	0.397	**<0.001**	**0.012**	0.573	**<0.001**
ballMPC:LTR - ballMPC:STR	0.445	0.514	0.88	0.451	0.82	**0.028**
(Control:STR - Control:BL) - (eeMPC:STR - eeMPC:BL)		0.067		0.156		
(Control:LTR - Control:BL) - (eeMPC:LTR - eeMPC:BL)		**0.02**		**0.01**		
(Control:LTR - Control:STR) - (eeMPC:LTR - eeMPC:STR)		0.643		0.237		
(Control:STR - Control:BL) - (HG:STR - HG:BL)		0.469		0.88		
(Control:LTR - Control:BL) - (HG:LTR - HG:BL)		0.263		0.156		
(Control:LTR - Control:STR) - (HG:LTR - HG:STR)		0.644		0.176		
(Control:STR - Control:BL) - (ballMPC:STR - ballMPC:BL)		0.643		0.156		
(Control:LTR - Control:BL) - (ballMPC:LTR - ballMPC:BL)		0.547		0.585		
(Control:LTR - Control:STR) - (ballMPC:LTR - ballMPC:STR)		0.324		0.336		
(eeMPC:STR - eeMPC:BL) - (HG:STR - HG:BL)		**0.009**		0.147		
(eeMPC:LTR - eeMPC:BL) - (HG:LTR - HG:BL)		**<0.001**		**<0.001**		
(eeMPC:LTR - eeMPC:STR) - (HG:LTR - HG:STR)		0.469		**0.014**		
(eeMPC:STR - eeMPC:BL) - (ballMPC:STR - ballMPC:BL)		**0.02**		**0.004**		
(eeMPC:LTR - eeMPC:BL) - (ballMPC:LTR - ballMPC:BL)		0.093		**0.002**		
(eeMPC:LTR - eeMPC:STR) - (ballMPC:LTR - ballMPC:STR)		0.547		0.83		
(HG:STR - HG:BL) - (ballMPC:STR - ballMPC:BL)		0.643		0.176		
(HG:LTR - HG:BL) - (ballMPC:LTR - ballMPC:BL)		0.067		0.337		
(HG:LTR - HG:STR) - (ballMPC:LTR - ballMPC:STR)		0.15		**0.026**		

*The multiple comparisons accounted for changes of metrics from baseline (BL) to short-term retention (STR) and long-term retention (LTR). The white-shaded rows are within-group differences, and gray-shaded rows are the interaction contrasts (between-group differences), performed when there was a significant Group x Time interaction as a result of ANOVA. Significant p-values are indicated in bold font*.

The changes in the answers from the questionnaires (intrinsic motivation and agency) were analyzed using the LME in Equation 8, with *Time* points: baseline, training, and long-term retention. Furthermore, the association between the changes in the sense of agency from baseline to training and the hitting task performance from baseline to short-term retention was investigated with Pearson correlation.

## 3. Results

We did not find significant differences between training groups during baseline in any of the metrics considered.

We found a significant correlation between the hitting performance (*score*) and the deviation from the pendulum natural frequency (*PSD % around* ω_*n*_, repeated-measures correlation, *r* = −0.59, *p* < 0.001), supporting the idea that mastering the hitting task is, in part, associated with better control of the pendulum dynamics.

### 3.1. Robot-Human Interaction and Assisting Forces During Training

**Figure 5:**
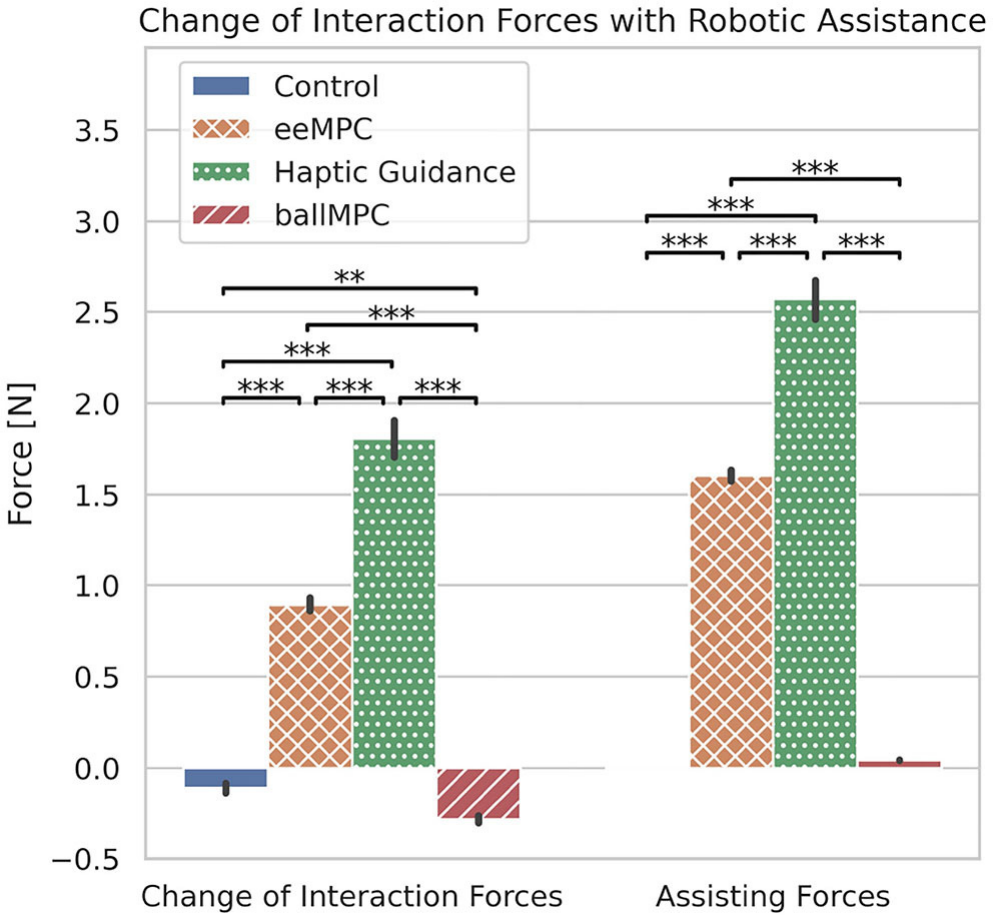
Differences between Assistance *ON* and *OFF* in the estimated human-robot interaction forces and applied assisting forces—both in *y* axis—for each training strategy. The error bars indicate 95% confidence interval. ^**^p < 0.01, ^***^p < 0.001.

When the assisting forces were turned on during the training blocks, the **interaction forces** between the robot and the participants increased significantly, compared to the catch-trials blocks, in the groups eeMPC and HG, but decreased significantly in the Control and ballMPC groups (Figure 5, Table 1). We found a significant interaction effect between the training group and trials with and without assistance on the interaction forces [*Group* *x* *Assistance*_*ON*/*OFF*_: *F*_(3,1200)_ = 661.2, *p* < 0.001]. *Post-hoc* comparison (Table 1, gray-shaded rows) revealed that the HG group increased the interaction forces significantly more than the other groups. The interaction forces also increased significantly more in the eeMPC group compared to the Control and ballMPC groups. We also found that training with ballMPC reduced the interaction forces to an even greater extent than the Control group.

We also found a significant interaction effect between the training group and trials with and without assistance on the applied **assisting forces** [Figure 5, *Group* *x* *Assistance*_*ON*/*OFF*_: *F*_(3,1200)_ = 1321.9, *p* < 0.001]. The *post-hoc* comparison (Table 1, gray-shaded rows) revealed that the assisting forces applied by the haptic guidance controller were significantly higher than those applied by the other training strategies. The assisting forces in the eeMPC group were also significantly higher compared to the Control and ballMPC groups.

### 3.2. Performance During Training With Different Training Strategies

**Figure 6:**
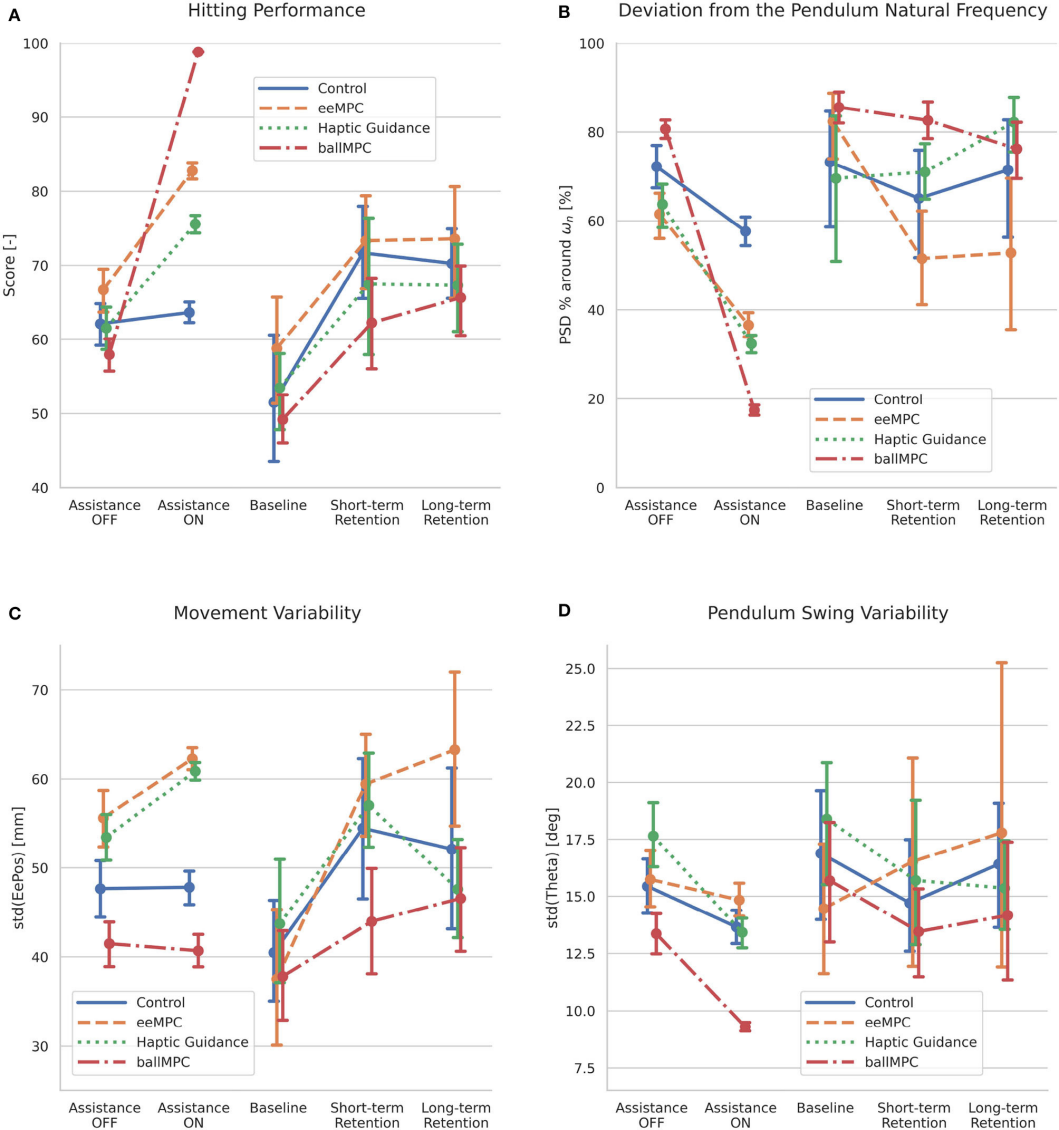
Effect of the training strategies on participants' performance during training (assisting forces *ON* and *OFF*) and baseline and retention tests. **(A)** Hitting performance (*score*). **(B)** Deviation from pendulum natural frequency (*PSD* % *around* *w*_*n*_). **(C)** Movement variability [*std*(*EePos*)]. **(D)** Pendulum swing variability [*std*(*Theta*)]. Error bars indicate the 95% confidence interval.

All participants (except those in the Control group) significantly increased their **hitting performance**, *score*, when the assisting forces were applied during training (Figure 6A, Table 1). We found a significant interaction effect between the training group and the application of the assisting forces [*Group* *x* *Assistance*_*ON*/*OFF*_: *F*_(3,1200)_ = 299.4, *p* < 0.001]. In particular, the increase of the *score* when the assistance was applied was significantly higher in the ballMPC group compared to all the other training groups (Table 1, gray-shaded rows). The application of eeMPC also increased the task performance significantly more than the Control group, and more than the HG group, although the difference did not reach significance (*p* = 0.09). Finally, the assisting forces from HG increased the score significantly more than the Control group.

All participants increased the **deviation from the pendulum natural frequency** (i.e., the *PSD % around* ω_*n*_ decreased) when the assisting forces were introduced during training (Figure 6B, Table 1). The Control group also reduced the *PSD % around* ω_*n*_ with respect to the catch-trials, even if there were no differences in the control strategy between those blocks/trials. We found a significant interaction effect between the training group and the addition of the assisting forces [*Group* *x* *Assistance*_*ON*/*OFF*_: *F*_(3,1200)_ = 97.9, *p* < 0.001]. *Post-hoc* comparison (Table 1, gray-shaded rows) revealed that the reduction in the ballMPC group was significantly larger than all the other groups. The *PSD % around* ω_*n*_ reduction was significantly larger in the HG group, compared to eeMPC and the Control groups. Finally, the reduction of the eeMPC group was significantly higher compared to Control.

The **performance variability**, *std*(*score*), decreased when the assistance was turned on in all groups except Control (Supplementary Figure 1a, Table 1). We found a significant interaction between the training group and the addition of the assisting forces [*Group* *x* *Assistance*_*ON*/*OFF*_: *F*_(3,1200)_ = 354.9, *p* < 0.001]. In particular, the decrease in the ballMPC group was significantly larger than in other groups (Table 1, gray-shaded rows). The eeMPC group reduced the performance variability significantly more than the Control. Finally, the decrease associated with the HG was significantly larger compared to Control.

The **movement variability**, *std*(*EePos*), significantly increased only when the assisting forces were applied in the eeMPC and HG groups (Figure 6C, Table 1). We found a significant interaction between the training group and the application of the assisting forces [*Group* *x* *Assistance*_*ON*/*OFF*_: *F*_(3,1200)_ = 21.3, *p* < 0.001]. *Post-hoc* comparison (Table 1, gray-shaded rows) revealed that the addition of the assisting forces in the HG and eeMPC groups increased the movement variability to a significantly greater extent than the ballMPC and Control groups.

The **pendulum swing variability**, *std*(*Theta*), significantly decreased in all groups, except eeMPC, when the assisting forces were applied (Figure 6D, Table 1). We found a significant interaction between the training group and the application of the assisting forces [*Group* *x* *Assistance*_*ON*/*OFF*_: *F*_(3,1200)_ = 11.9, *p* < 0.001]. In particular, the HG and ballMPC groups decreased the pendulum swing variability significantly more than the eeMPC group (Table 1, gray-shaded rows). The variability decrease was significantly higher in the HG and ballMPC groups compared to the Control group.

### 3.3. Effect of the Training Strategies on Motor Learning

All participants improved their **hitting performance** (*score*) from baseline to short- and long-term retention (Figure 6A, Table 2). We did not find differences across groups.

Only participants who trained the complex pendulum task with eeMPC increased significantly the **deviation from the pendulum natural frequency** (i.e., the *PSD % around* ω_*n*_ significantly decreased) from baseline to short-term (Figure 6B, Table 2) and long-term retention. We found a significant interaction between the training group and *Time* [*Group* *x* *Time*: *F*_(6,80)_ = 4.71, *p* < 0.001]. In particular, participants in the eeMPC group decreased the *PSD % around* ω_*n*_ significantly more than the ballMPC and HG groups at short-term retention (Table 2, gray-shaded rows), while the difference with the Control group did not reach significance (*p* = 0.07). The eeMPC group also decreased the *PSD % around* ω_*n*_ significantly more than the HG and Control groups at long-term retention, while the difference with the ballMPC group did not reach significance (*p* = 0.09).

All participants reduced the **performance variability**, *std*(*score*), significantly from baseline to short-term retention (Supplementary Figure 1a, Table 2) and long-term retention. We did not find significant differences between groups.

All groups, except the ballMPC, increased the **movement variability**, *std*(*EePos*), significantly from baseline to short-term retention (Figure 6C, Table 2), while all groups, except HG, increased the movement variability significantly from baseline to long-term retention. HG reduced the movement variability significantly from short-term to long-term retention. We found a significant interaction between training group and *Time* [*Group* *x* *Time*: *F*_(6,80)_ = 6.14, *p* < 0.001]. In particular, the eeMPC group increased the movement variability significantly more than the ballMPC at short-term retention, and significantly more than all the other groups at long-term retention (Table 2, gray-shaded rows). The increase of the movement variability from short-term to long-term retention was significantly higher with eeMPC compared to HG and higher with ballMPC compared to HG.

None of the participants changed the **pendulum swing variability**, *std*(*Theta*), significantly from baseline to short-term retention, from baseline to long-term retention or between retention blocks (Figure 6D, Table 2). Although the eeMPC group tended to increase the pendulum swing variability at short-term, while the other groups tended to reduce it, we did not find significant differences between groups [*Group* *x* *Time*: *F*_(6,80)_ = 1.6, *p* = 0.15].

### 3.4. Effect of the Training Strategies on Transfer

We evaluated the effect of the different training strategies on the **transfer task performance** (i.e., learning to invert the pendulum, *score*_*transfer*_). All groups, except the ballMPC, improved significantly their performance from transfer baseline to short-term retention (Supplementary Figure 1b, Table 2). All groups improved significantly the score from transfer baseline to long-term retention. Only the eeMPC and ballMPC groups improved significantly the score from short-term to long-term retention. However, the (*Group* *x* *Time*) interaction only approached significance [*F*_(6,80)_ = 2.05, *p* = 0.069].

### 3.5. Effect of Training Strategies on Agency and Motivation

**Figure 7:**
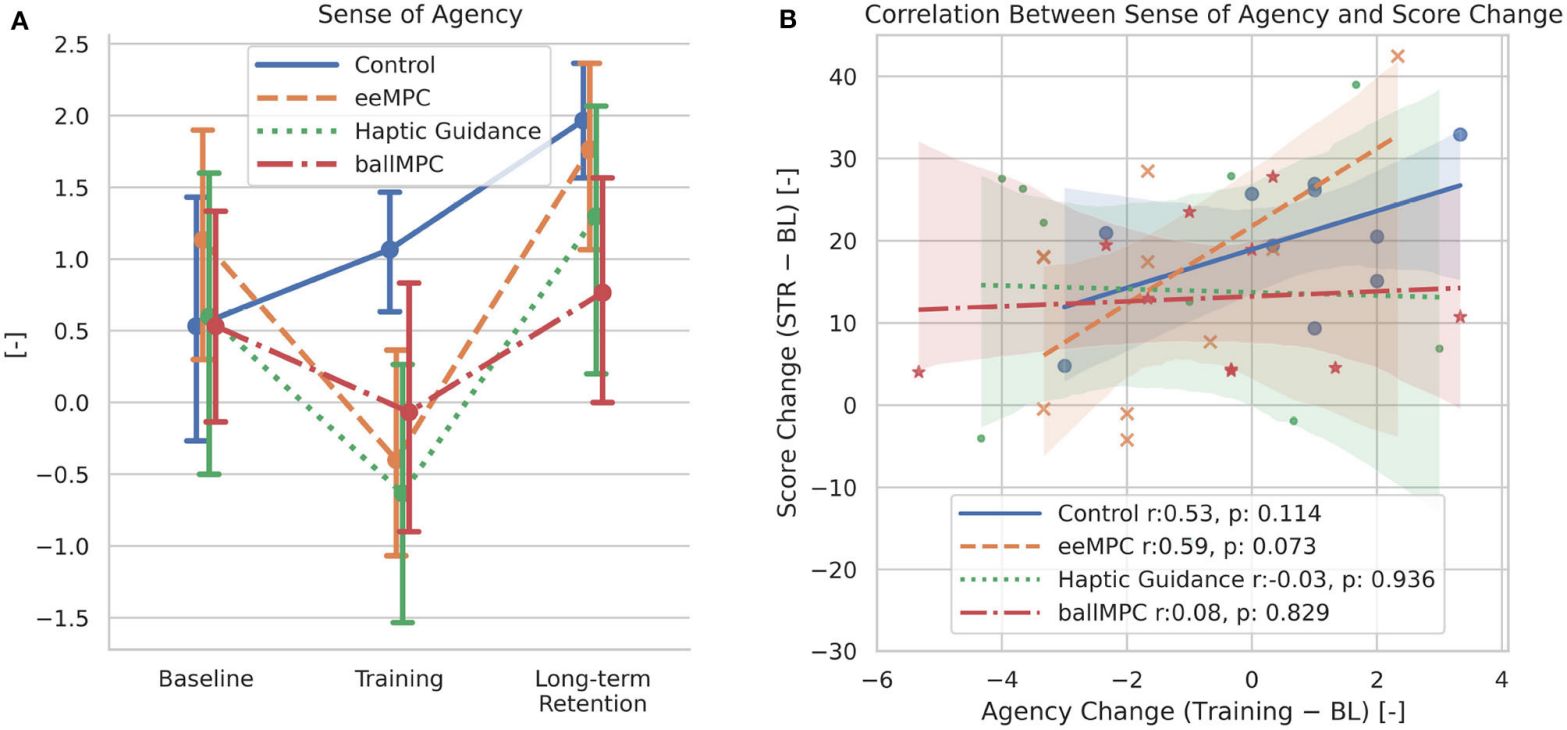
**(A)** Effect of the training strategy on changes in responses to the agency questionnaire from baseline to after training and long retention test. **(B)** Association between the change of sense of agency and *score* increase from baseline to short term retention. Error bars indicate the 95% confidence interval.

Participants in the eeMPC and HG groups reduced their sense of **agency** during training, although the reduction in HG did not reach significance (Figure 7A, Supplementary Table 1). The sense of agency in these two groups increased significantly from training to the second experimental session. However, we did not find a significant interaction effect between the training group and *Time*.

In order to evaluate the association between the sense of agency during training and the improvement in the hitting task accuracy (*score*) after training, we performed correlations between the changes of agency and task performance from baseline to short-term retention. When taking all groups together, we only found a one-sided significant association (Figure 7B, Pearson correlation, *r* = 0.262, *p* = 0.101). Interestingly, when analyzing the correlation within each training group, we observed a positive association between the sense of agency and amount learned, especially in the eeMPC group (eeMPC: *r* = 0.59, *p* = 0.07).

The overall mean **interest/enjoyment** remained high (~5.5) for all the groups during the whole experiment. None of the training groups reported significant differences in their interest/enjoyment from baseline to training, or from training to the second experimental session (Supplementary Figure 2a). We did not find significant interaction effects between the training groups and *Time*.

All participants increased their **perceived competence** from baseline to training, although only the HG and ballMPC groups did it significantly (Supplementary Figure 2b, Supplementary Table 1). All participants increased their perceived competence from baseline to long-term retention. The interaction between training group and *Time* did not reach significance (*Group* *x* *Time*: *p* = 0.11). However, it is interesting to note that while participants who trained with ballMPC reduced their perceived competence from training to the second experimental session, the others groups reported higher competence levels during the second session.

In general, participants reported higher levels of **effort/importance** after training, however differences only approached significance in the Control, eeMPC and HG groups (Supplementary Figure 2c, Supplementary Table 1). The eeMPC reduced the effort/importance again from training to the second session, although did not reach significance. It is interesting to note that the ballMPC group seemed to report systematically lower levels of effort/importance, compared to the other groups. However, we did not find significant interaction effects between the training groups and *Time*.

None of the training strategies changed the self-reported **pressure/tension** significantly from baseline to after-training measurements and only in the eeMPC significantly decreased from baseline to long-term retention (Supplementary Figure 2d, Supplementary Table 1) and from training to long-term retention. However, we did not find a significant interaction effect between the training group and *Time*.

## 4. Discussion

### 4.1. Training With MPCs Reduced the Assisting Forces and Increased Task Performance During Training

According to the Guidance Hypothesis, haptic guidance might disrupt motor learning because participants rely on the assisting forces, limiting the learning of correct muscle activation patterns required to perform the task in the absence of assistance (Schmidt and Bjork, 1992). Therefore, we hypothesized that MPCs could be better training strategies, as they minimize the assisting forces in real-time based on participants' ongoing performance. Furthermore, minimizing assisting forces using MPCs might also reduce the interference between the assisting forces and the haptically rendered dynamics, supporting the learning of dynamic-dependent tasks (Powell and O'Malley, 2012; Pezent et al., 2019).

In line with our expectations, the assisting forces of both the MPCs, regardless of the point of application (end-effector [eeMPC] or pendulum ball [ballMPC]), were significantly lower compared to haptic guidance. Furthermore, training with both MPCs resulted in significantly less human-robot interaction forces than training with haptic guidance. When the assisting forces were applied at the pendulum ball, the interaction forces were even smaller than during non-assisted trials. These smaller assisting forces did not come at the cost of task performance. In fact, training with both the MPCs resulted in a higher increase in task performance (*score*) during training compared to training with haptic guidance. We also observed a significantly higher task performance in the ball MPC, compared to the other strategies. This is probably due to the fact that the assisting forces applied to the pendulum ball could be less counteracted by the participant, in contrast to the groups where assisting forces were provided directly at the robot end-effector. Therefore, the MPCs accomplished the aim of minimizing both the assistance and interaction forces without degrading task performance.

### 4.2. Training With the End-Effector MPC Decreased the Performance Variability, Increased the Movement Variability, and Did Not Hamper the Pendulum Swing Variability During Training

An important limitation associated with haptic guidance is that physically constraining the movement to a fixed trajectory might reduce motor variability (Duarte and Reinkensmeyer, 2015; Ivanova et al., 2020). We hypothesized that the end-effector MPC would overcome this problem as the optimal trajectory is recalculated in real-time, and therefore, allows for multiple trajectory solutions—i.e., better respecting the participants' natural motor variability, which is necessary for learning (Dhawale et al., 2017).

In this paper, we distinguished between three different types of motor variability: (i) performance variability, i.e., variability in the task score, normally associated to low expertise in the motor task (Harris and Wolpert, 1998), (ii) movement variability, i.e., the variability of the participant's movement, a feature that might promote motor learning (Dhawale et al., 2017), and (iii) pendulum swing variability, i.e., the variability of the pendulum angle, which might be especially important to promote the exploration of the task dynamics (Muller and Sternad, 2004). We hypothesized that the assisting forces from the end-effector MPC would increase the movement and pendulum swing variability during training while limiting the frustrating performance variability (Duarte and Reinkensmeyer, 2015). On the other side, we expected that the assistance from the ball MPC would reduce not only the performance variability but also the movement and swing variability. Since the assisting forces from the ballMPC, albeit being lower, directly act on the pendulum ball, the ball would move accurately with only minimal input from the participants, and therefore, they would not feel prompted to explore new movement trajectories, limiting the movement and pendulum swing variability.

As expected, participants significantly reduced their performance variability when the robotic assistance was applied. Our finding on increased task performance during training with MPC-based robotic assistance is consistent with previous research on the use of more conventional robotic training strategies in motor learning (Marchal-Crespo and Reinkensmeyer, 2008; Marchal-Crespo et al., 2017a). Both MPCs reduced the performance variability during training significantly, although the reduction in the ball MPC was significantly more pronounced than both the end-effector MPC and haptic guidance. On the other side, the movement variability increased when the assisting forces from the haptic guidance and end-effector MPC were applied, while it was not affected by the ball MPC. The increase in movement variability may be explained by the kinematic requirements of the complex dynamic task. In order to successfully hit the targets while compensating for the natural swing of the pendulum, large and variable end-effector movements are needed. Therefore, applying assistance at the end-effector to fulfill the hitting task results in more variable movements. On the other side, when the assisting forces are applied to the pendulum ball, participants hardly need to move to achieve the task.

The fact that we could not detect a significant difference in the movement variability between the end-effector MPC and haptic guidance, despite the flexible trajectories promoted by the MPC, might be due to the variable location of the targets. The movement variability at each block was calculated from the trajectories performed between two consecutive targets, and the targets were located at different locations within a block—i.e., the diversity of trajectories between different targets increased the inherent variability, even if the participants would follow them without a deviation due to the inflexible HG trajectory calculation. Thus, the participants' trajectory freedom associated with training with the end-effector MPC might have been masked by the default high movement variability associated with skillful task performance—i.e., the metric was not sensitive enough to detect differences between groups.

Importantly, the end-effector MPC did not hamper the pendulum swing variability when the assisting forces were applied, contrary to the haptic guidance and ball MPC strategies. This might be a result of the flexible trajectories promoted by the end-effector MPC. In fact, the flexibility in the end-effector trajectories might explain both, the increase in the end-effector movement variability and the swing variability. Therefore, the participants' freedom of movement promoted by the end-effector MPC was more apparent within the pendulum swing variability.

### 4.3. Training With the End-Effector MPC Enhanced Learning of the Complex Dynamic Task

Based on the idea that the end-effector MPC minimizes the assisting forces depending on the participant's ongoing performance while promoting flexible trajectories that increases movement and pendulum swing variability, we hypothesized that training with the end-effector MPC would result in better motor learning of the complex dynamic task. Our results only partially confirm our hypothesis.

We quantified the learning of the complex dynamic task using a metric directly linked to task success (i.e., the target hitting performance [*score*]), and a second metric related to the control of the pendulum dynamics (i.e., the deviation from the pendulum natural frequency [*PSD % around* ω_*n*_]). We found a significant correlation between both performance metrics, supporting the idea that task success is associated, in part, with better control of the pendulum dynamics. However, this correlation does not imply a functional relation such that participants with a larger deviation from the pendulum natural frequency would achieve higher scores. Other ways of achieving higher scores might exist in this specific chaotic system, besides increasing the deviation from the pendulum natural frequency.

Only participants who trained the complex dynamic task with the end-effector MPC increased significantly the deviation from the pendulum natural frequency at short- and long-term retention, and significantly more than the other groups. This means that the end-effector MPC group got better at controlling the pendulum swing movement by driving it away from the frequency which the pendulum would naturally swing without intervention—i.e., they learned better how to control the dynamics of the pendulum. We also found that training with the end-effector MPC resulted in significantly more variable movements at long-term retention compared to the haptic guidance group. Interestingly, although training with haptic guidance increased the movement variability at short-term retention, the benefit disappeared at long-term retention. Thus, the benefit of haptic guidance on movement variability seems to be only transitory, highlighting the need to consistently test for long-term retention in motor learning studies (Williams and Carnahan, 2014). Overall, training with the end-effector MPC resulted in more variable movements and better learning to control the pendulum dynamics.

A potential rationale behind the superiority of the end-effector MPC strategy may be the significantly lower forces applied during training, compared to haptic guidance. Minimizing the assisting forces probably reduced the potential slacking effect observed in previous studies with haptic guidance (Schmidt and Bjork, 1992; Marchal-Crespo and Reinkensmeyer, 2008). Furthermore, the smaller assisting forces probably allowed participants to gain a better understanding of the pendulum dynamics, as the potential interference of the assisting forces with the haptically rendered dynamics was reduced (Powell and O'Malley, 2012). Furthermore, increasing the movement variability while not hampering the swing variability likely encouraged the exploration of the pendulum dynamics (Muller and Sternad, 2004).

However, training with lower assisting forces did not help participants trained with the ball MPC to learn how to control the pendulum dynamics. Although the assisting forces were significantly lower than both the end-effector MPC and haptic guidance strategies, the application point of the assistance probably deteriorated the perception of the task dynamics. The assistance on the ball corrupted the natural oscillation of the pendulum (i.e., the magnitude of the gravitational forces that drive the natural oscillation—in Equation (1)—were smaller than the magnitude of the assisting forces), and probably hampered the learning of the dynamic task.

Literature suggests that the amount and consistency of errors during training modulate motor learning (Herzfeld et al., 2014). Therefore, considering that the groups experienced different errors during training, differences between groups in the hitting performance and performance variability at short- and long-term retention tests should be observed. In particular, we hypothesized that the higher movement and pendulum swing variability during training with the end-effector MPC would result in better hitting performance, while the limited movement variability and higher scores observed during training with the ball MPC would hamper learning of the hitting task. However, while all participants learned the task (i.e., all groups increased the *score* and reduced the performance variability significantly), we did not find significant differences between groups.

Taking together, although training with the end-effector MPC enhanced learning to control the pendulum dynamics (measured by the deviation from the pendulum natural frequency), this was not reflected in a more accurate hitting performance (measured by *score*) when compared to the other training strategies. Although the variability in the pendulum swing frequency accounted for most of the variability in the target hitting performance (the correlation coefficient was *r* = −0.59), due to the chaotic nature of the task dynamics, there were probably other factors as well—e.g., transient pendulum movements after hitting the previous targets—that affected the target hitting performance. Thus, the *score* metric, although correlated with the deviation from the pendulum natural frequency, was probably not sensitive enough to fully capture the learning of the complex dynamic task. For tasks/environments that are complex (e.g., non-linear, under-actuated) and redundant (i.e., allow multiple movement solutions to achieve the same result), it may not be sufficient to analyze the end-result performance to fully capture the learning process. Detailed kinematic analysis of such systems (e.g., movement of the end-effector, pendulum swing frequency, etc.) may carry important information regarding how participants' movements improve with learning (Zhang et al., 2018; Bazzi and Sternad, 2020).

### 4.4. The Benefit of the End-Effector MPC on Learning the Dynamic Task Did Not Generalized to the Transfer Task

We expected that better control of the pendulum dynamics gained during training would result in better performance in the transfer task—i.e., inverting the pendulum. Therefore, we anticipated that training with the end-effector MPC, which enhances the control of the pendulum dynamics and does not hamper the pendulum swing variability during training, would enhance transfer learning. However, while all training groups improved their performance in the transfer task, we did not find significant differences between strategies. These unexpected results may derive from the substantial differences between the training and transfer tasks. During the execution of the target-hitting task, the experienced pendulum angles were mostly around the stable equilibrium point (i.e., θ = 0 °). On the other side, the aim of the transfer task shifted the experienced angles toward the unstable equilibrium point (i.e., inverted pendulum configuration θ = 180 °). In literature, differences in brain activation, especially in the cerebellar activity, were observed depending on whether the experienced complex manipulation dynamics were around the stable or unstable states of a dynamic system (Milner et al., 2006). Thus, a transfer task that requires exploration of similar pendulum states as the target-hitting task, such as the same task with slightly different pendulum dynamics (e.g., different mass or gravity coefficient), or with different target locations, might have been more sensitive to detect differences in transfer between the training strategies.

### 4.5. Agency and Motor Learning Might Be Associated, Especially When Training With the End-Effector MPC

We hypothesized that training with the ball MPC would negatively affect the sense of agency due to the indirect application point of the assisting forces, compared to the end-effector MPC. Lower agency associated with indirect application point of assisting forces was observed on a preliminary experiment with a similar task (Özen et al., 2019). In this study, we observed a decrease in the agency when the participants were assisted—compared to when they were not assisted—in all assistance strategies. However, this decrease in the agency did not significantly differ between groups; neither during training nor at long-term retention.

Importantly, we observed a positive association between the change of sense of agency and *score* from baseline to short-term retention. When analyzing the correlation within each training group, we observed a positive association between the sense of agency and amount learned, especially in the end-effector MPC group. However, we note that the sample size was small, and the correlations above *r* > 0.5 depend on only a few data points. Such correlations with small sample sizes can be variable across samples, therefore, the interpretation of this finding should be taken cautiously.

A rationale behind the observed association could be the increase in the predictability of the pendulum motion when training with the end-effector MPC. Literature suggests that high levels of agency are associated with high predictability in the human-robot interaction forces (Ivanova et al., 2020). Furthermore, it was shown on a similar pendulum task that participants adapt their movements to achieve a more predictable pendulum motion (Maurice et al., 2018; Bazzi and Sternad, 2020). Since the end-effector MPC group experienced more flexible trajectories—especially compared to haptic guidance—and higher pendulum swinging variability, participants who better mastered the task may have been able to make their movements more predictable during the training, which increased their sense of agency. Alternatively, the adaptability of the assistance from the end-effector MPC might have played a role in the sense of agency. As the assisting forces from the MPC are optimized based on participants' performance, more skilled participants required less assisting forces, and therefore, participants might have felt more in control of their movements. Although the haptic guidance forces were also smaller in the participants who performed better, the fixed trajectories enforced by the controller probably hampered their sense of agency. On the other side, the unpredicted change of perceived dynamics when training with the ball MPC may have affected the sense of agency negatively by decreasing the congruency between the participants' movements and the pendulum movement (Endo et al., 2020).

To conclude, an increase in the sense of agency might be an indication of a more accurate prediction of the consequences of participants' actions, and therefore, reflect motor learning (van der Wel et al., 2012). Furthermore, the sense of agency has been shown to be fundamental for interacting with the external world (Haggard and Tsakiris, 2009), and rely on brain areas associated with motor control (Chambon et al., 2012). Therefore, robotic assistance strategies that lead to increases in agency might be beneficial for motor learning.

### 4.6. No Differences in Motivation Across Training Strategies

Although previous research has shown that motivation can be modulated through robotic assistance (Duarte and Reinkensmeyer, 2015; Marchal-Crespo et al., 2017b; Bernardoni et al., 2019), we did not find significant differences between training strategies in any subscale of the Intrinsic Motivation Inventory. The lack of significant differences could be due to the generally high overall interest/enjoyment and effort/importance (i.e., ceiling effect) and the high variability observed across participants.

We found a significant increase in the perceived competence in all training groups after training and at long-term retention. However, while participants who trained with the ball MPC reported lower levels of perceived competence from training to the second experimental session, all the other groups reported higher competence levels (although the *score* after training with the ball MPC was at the same level as the other groups). This might be due to the high performance that the ball MPC group experienced during training. Probably, when they returned for the second session to test long-term retention, the performance degradation resulted in a decrease in the perceived competence. Therefore, caution should be put when designing training strategies that result in almost perfect performance during training, as it might have a negative long-lasting effect on participants' perceived competence when the assistance is removed.

### 4.7. Study Limitations

The experimental design suffers from several limitations. First, although the experiment was not excessively long (the first session was around 30 min), the task was quite repetitive, and therefore, it could potentially promote participants' lack of attention, especially toward the end of the session. However, we note that participants' interest/enjoyment and effort/importance, as measured with the IMI questionnaire, were systematically high during the whole experiment duration.

Secondly, participants were randomly allocated to one of the four control strategies (parallel design). The random allocation of participants into different training groups might result in differences in baseline performance between groups—especially in small population samples—that might bias the results—e.g., participants' initial skill level has been shown to play an important role in the effectiveness of different haptic methods (Marchal-Crespo et al., 2010, 2013, 2017a; Duarte and Reinkensmeyer, 2015). In order to control for the personal abilities to perform the task, we added a random intercept for the *Subject* and a random slope for the *Time* in the linear mixed model. We further confirmed that there were no differences in the baseline performance between training groups.

Finally, the randomized location of the targets might have had an unforeseen effect on the results. Although the randomization kept the task challenging and motivating, it made the comparison of the movement variability between the training groups difficult, as discussed above. Furthermore, the differences in the order of the target locations between training and catch-trial blocks could have affected participants' movement and performance to a certain degree. This is supported by the significant, albeit small, differences observed in the human-robot interaction forces and deviation of the pendulum natural frequency in the control group between the training and catch-trail blocks, even though no robotic assistance was provided during the training blocks.

## 5. Conclusion

We showed that training with novel robotic assistance strategies based on Model Predictive Control enhances the learning of a complex dynamic task. Training with the novel MPC strategy (end-effector MPC) resulted in a significant reduction of the assisting forces and more flexible trajectories when compared to a conventional haptic guidance approach. Furthermore, training with the MPC strategy resulted in higher task expertise, reducing the amount of errors and performance variability during training, and better control of the complex dynamic system, reflected in enhanced movement and pendulum swing variability. Further, training with the novel MPC approach, especially when participants are more skilled, seems to improve the sense of agency. Attention should be given to the application location of the assisting forces, since this location may affect the perceived dynamics of the environment and the sense of agency.

Together, Model Predictive Controllers may be beneficial for neurorehabilitation, since they could outperform haptic guidance by reducing assisting forces and increasing the movement variability while still keeping brain-injured patients' motivation and engagement high. Their benefits for the recovery of stroke patients should be evaluated in a future clinical study.

## Data Availability Statement

The dataset presented in this study can be found online in the following repository: http://doi.org/10.5281/zenodo.4390063.

## Ethics Statement

The studies involving human participants were reviewed and approved by Cantonal Ethics Committee and the Swiss Agency for Therapeutic Products (Swissmedic). The patients/participants provided their written informed consent to participate in this study.

## Author Contributions

ÖÖ contributed to the virtual training environment, robotic assistance strategies, study design, data acquisition, data analysis, and writing the manuscript. KB contributed to the study design, preparation of the questionnaires, data analysis, and revising the manuscript. LM-C supervised the project, contributed to the study design, data analysis, and writing the manuscript. All authors contributed to the article and approved the submitted version.

## Conflict of Interest

The authors declare that the research was conducted in the absence of any commercial or financial relationships that could be construed as a potential conflict of interest.
