# Supplementary material for: Promoting Motor Variability During Robotic Assistance Enhances Motor Learning of Dynamic Tasks
Source: Front Neurosci. 2021 Feb 2;14:600059. doi: 10.3389/fnins.2020.600059 (PMC7884323; doi:10.3389/fnins.2020.600059)
Supplement: Supplementary file 1 [file Data_Sheet_1.pdf]

Supplementary Material

1 IMPLEMENTATION DETAILS OF THE TRAINING STRATEGIES

1.1 End-effector MPC (eeMPC)

The eeMPC was implemented using ACADO Toolkit (Houska et al., 2011), similar to (Özen et al., 2019), but with some simplifications to increase its sampling frequency, and therefore, performance. In contrast to the two-dimensional control input of (Özen et al., 2019), eeMPC could apply its assisting forces (control force) only on the y axis. The eeMPC was formulated as sparse Quadratic Programming (fully condensing). The differential equation of the eeMPC contained a non-accelerating pendulum pivot point:

$$\ddot{\theta} = -\frac{1}{l}\left(\frac{F_y}{M_{ee}}\cos\theta + g\sin\theta\right) - \frac{c}{ml^2}\dot{\theta}. \tag{S1}$$

The θ, m, l, and c are the pendulum angle, the mass of the pendulum red ball, the length of the pendulum rod, and the rotational damping, respectively. F_y is the assisting force on the pendulum. M_{ee} is the inertia of the end-effector of the robot which is approximated to be a mass of 1 kg. The cost function of the eeMPC, from which the optimum force values are calculated, is the following:

$$\sum_{k=t}^{t+N} h\left(x_k, F_{y_k}\right)^{\top} W_k h\left(x_k, F_{y_k}\right) \tag{S2}$$

where t is the current time, k is the time step along the horizon and N is the predicted horizon length (and the terminal state index). x_k is the state of the system. W_k is the cost matrix. The $h\left(x_k, F_{y_k}\right)$ matrix consists of the y distance to the reference target position (d_y), the y velocity of the pendulum ball (v_y), the relative z velocity of the pendulum ball (with respect to the end-effector, v_z^{ee}), and the assisting forces:

$$h\left(x_k, F_{y_k}\right) = \begin{bmatrix} d_y \\ v_y \\ v_z^{ee} \\ F_y \end{bmatrix}. \tag{S3}$$

W_k is a function of k such that the weighting gains increase when approaching the time to pass the targets. Thus, the eeMPC assisted more when getting close to hitting the targets, but was more flexible just after passing the previous target. In addition to the cost function S2, the following variables were limited with a box constraint to satisfy the workspace/safety limitations and to provide a smooth/continuous assisting force profile: the end-effector y position and velocity; pendulum angle and velocity; assisting forces and the time derivative of assisting forces. The eeMPC applied forces up to 8 N. The horizon length of the eeMPC was 80 steps, corresponding to 12.5 ms for each step. The Gauss–Newton algorithm was used for hessian approximation. Multiple-shooting was used as a discretization method. Furthermore, the Gauss-Legendre integrator of order 4 was used for predicting future states.

1.2 Ball MPC (ballMPC)

The implementation of the ballMPC was the same as eeMPC, except that the assisting forces were applied at the pendulum ball and were limited to 1 N. The differential equation of the ballMPC was the following:

$$\ddot{\theta} = -\frac{1}{l}\left(g \sin\theta - \frac{F_y}{m}\cos\theta\right) - \frac{c}{ml^2}\dot{\theta}. \tag{S4}$$

1.3 Haptic Guidance (HG)

The haptic guidance strategy was implemented as a conventional PD controller of the following form:

$$F_y = M_{ee}\bigg(K_p(y_{ref} - y) + K_d(\dot{y}_{ref} - \dot{y})\bigg) \tag{S5}$$

where K_p (100 N/m.kg) and K_d (20 N.s/m.kg) were the proportional (P) and derivative (D) gains of the critically damped PD controller. M_{ee} (1 kg) was the inertia of the robot end-effector. In order to extract the reference trajectory (y_{ref}), the optimization step of the eeMPC was performed each time a target was passed. A cubic spline was fitted to the predicted trajectory of the eeMPC optimization, and this spline was used as a reference. The assisting force magnitude was limited to 8 N.

2 QUESTIONNAIRE

Agency:

- It seemed like I was in control of the pendulum.
- It seemed as if the pendulum was controlling me.
- It seemed like I was causing the movements of the pendulum.

Interest/Enjoyment:

- I thought this activity was quite enjoyable.
- The task was fun to do.
- I would describe this activity as very interesting.

Perceived Competence:

- I was pretty skilled at this activity.
- I am satisfied with my performance at this task.
- I think I am pretty good at this activity.

Effort/Importance:

- I tried very hard on this activity.
- I put a lot of effort into this.
- It was important to me to do well at this task.

Pressure/Tension:

- I felt pressured while doing these.
- I was anxious while working on this task.

- I felt very tense while doing this activity.

3 RESULTS

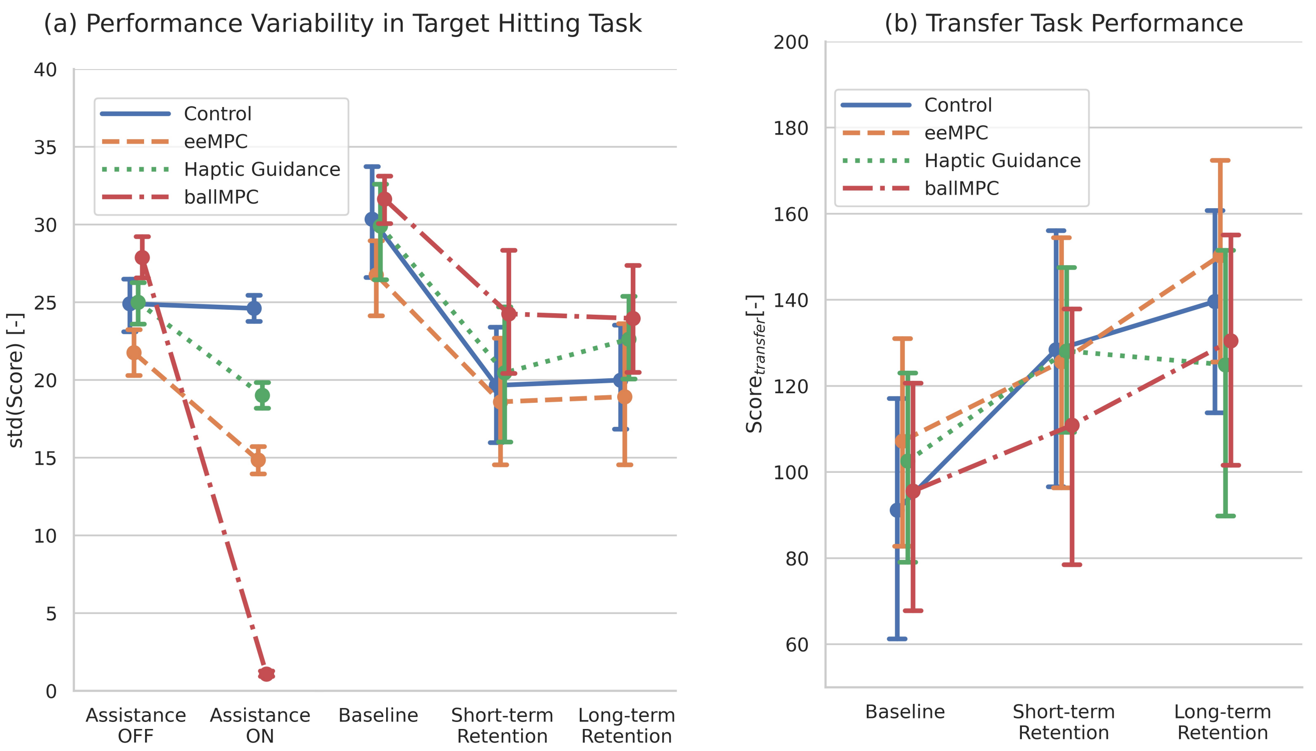

Figure S1: (a) Performance variability ($std(Score)$) of the target hitting task during training (assisting forces ON and OFF) and baseline and retention tests. (b) The transfer task performance ($Score_{transfer}$) during transfer baseline and transfer retention test. Error bars indicate the 95% confidence interval.

Table S1. Results from the multiple comparisons tests to evaluate the effects of the training strategies on after-training and long-term retention questionnaire responses. The multiple comparisons accounted for changes of metrics from baseline (BL) to after-training (T) and long-term retention (LTR). The values are within-group differences. There was no significant $Group\ x\ Time$ interaction found as a result of ANOVA. Significant p-values are indicated in bold font.

Comparison	Sense of Agency	Interest/Enjoyment	Perceived Competence	Effort/Importance	Pressure/Tension
Control:T - Control:BL	0.392	0.88	0.054	0.06	0.441
Control:LTR - Control:BL	**0.045**	0.671	**<0.001**	0.063	0.584
Control:LTR - Control:T	0.246	0.671	**0.04**	0.953	0.244
eeMPC:T - eeMPC:BL	**0.038**	0.672	0.087	0.476	0.918
eeMPC:LTR - eeMPC:BL	0.363	0.883	**0.006**	0.978	**0.028**
eeMPC:LTR - eeMPC:T	**0.004**	0.671	0.203	0.06	**0.028**
HG:T - HG:BL	0.086	0.81	**0.006**	0.095	0.53
HG:LTR - HG:BL	0.344	0.88	**<0.001**	0.803	0.3
HG:LTR - HG:T	**0.007**	0.88	0.331	0.241	0.077
ballMPC:T - ballMPC:BL	0.363	0.671	**<0.001**	0.545	0.584
ballMPC:LTR - ballMPC:BL	0.688	0.671	**0.037**	1.0	0.3
ballMPC:LTR - ballMPC:T	0.262	0.904	0.114	0.545	0.545

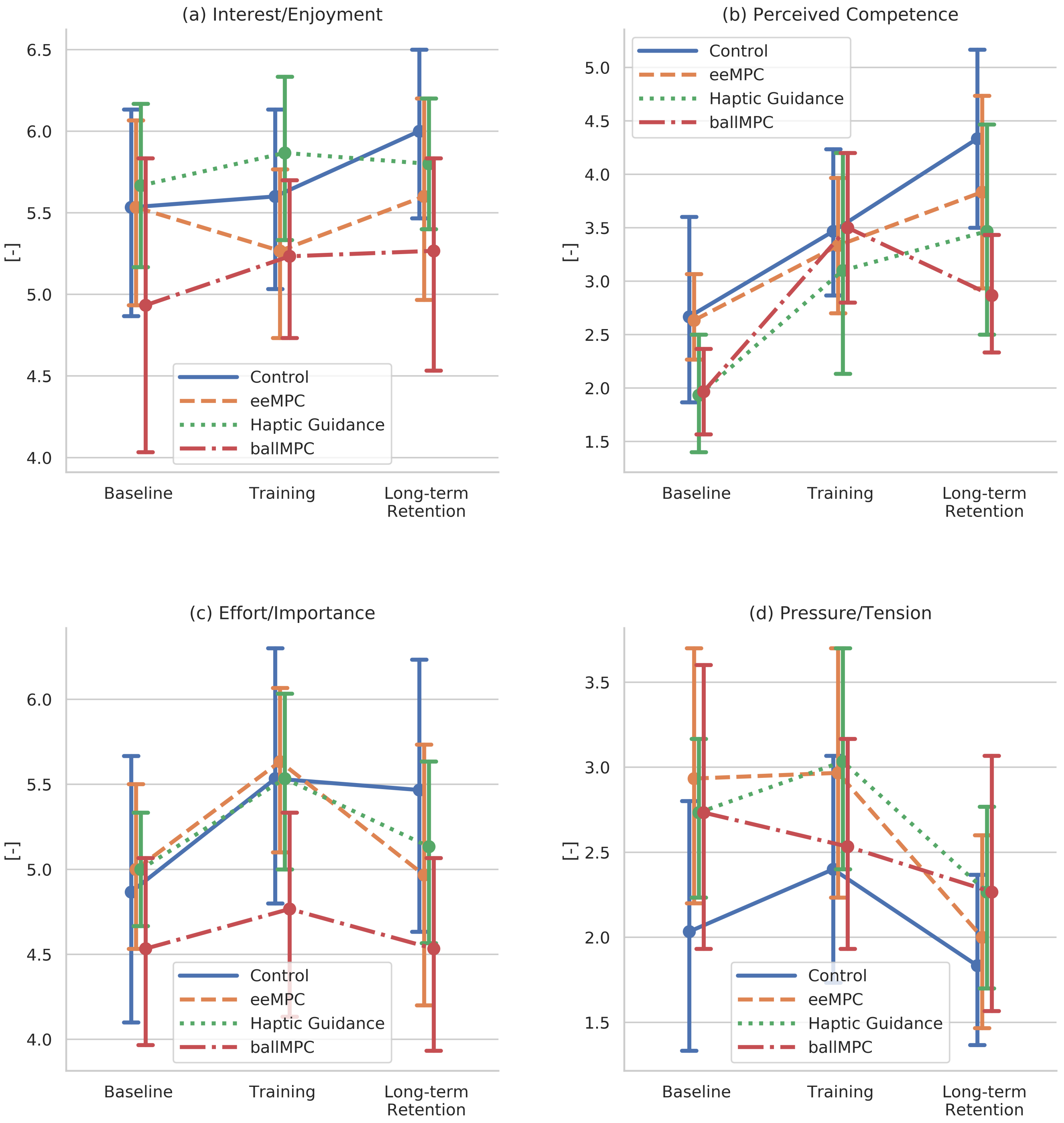

Figure S2: Effect of the training strategies on participants' self-reported values of intrinsic motivation after baseline, training and long-term retention: (a) *interest/enjoyment*, (b) *perceived competence*, (c) *effort/importance*, and (d) *pressure/tension*. Likert scales based on Ryan et al. (1990). The bars indicate the 95% confidence interval.

REFERENCES

Houska, B., Ferreau, H. J., and Diehl, M. (2011). ACADO toolkit-An open-source framework for automatic control and dynamic optimization. *Optimal Control Applications and Methods* 32, 298–312. doi:10.1002/oca.939. ZSCC: 0000692

Özen, O., Traversa, F., Gadi, S., Buetler, K. A., Nef, T., and Marchal-Crespo, L. (2019). Multi-purpose robotic training strategies for neurorehabilitation with model predictive controllers. In *2019 IEEE 16th International Conference on Rehabilitation Robotics (ICORR)*. 754–759. doi:10.1109/ICORR.2019.8779396

Ryan, R. M., Connell, J. P., and Plant, R. W. (1990). Emotions in nondirected text learning. *Learn. Individid. Differ.* 2, 1–17. doi:10.1016/1041-6080(90)90014-8
